# Anatomical Lucubrations at the Cape of Good Hope

**Published:** 1851-02

**Authors:** 


*Anatomical Lucubrations at the Cape of Good Hope.*— A writer in the African Journal, a periodical published at Cape Town, after quoting at full length a humorous extract from a recent number of the Lancet, in which a race of "men with tails" were *said* to have been discovered in Central Africa, reasons on the subject in such a guise that it is difficulty to discover whether he is in jest or in earnest. He says:

"The public do not know, as medical men and anatomists do, that every man and woman is born with and has a tail, composed of the five, six, or seven, fixed false vertebræ of the sacrum, and of the two, three, four, or five loose ones of the coccyx at its end, (in different individuals,) all of which, however, *in common men and women*, are covered by flesh and skin, and hidden;—so that it is *not at all impossible* that there may be a variety of men, even in Central Africa, forming a tribe, who all have a few more supplementary external joints to the os coccygis than are usual; in which case they would project, necessarily, beyond the skin, and form a pendulous and exterior tail; either short, as in the Manx cats, in rabbits, hares, or deer, or longer, as horses, dogs, monkeys, etc.; for *when once one child with this variety happened to have been born*, we know that the deformity would be perpetuated in a race like all the other varieties of men, as black, or colored, etc., or as in families with six toes, or six fingers, or two toes united, or fingers, or toes webbed;—or, as among swine in the solid-footed pigs of South America, &c."—He also copies a paragraph from another number of our journal, in which an improvement upon the foregoing configuration was instanced—an eye being implanted at the end of the tail. The learned writer thinks that one at the end of the forefinger would be better—at any rate for midwifery practitioners. He adds, however, "It will be a long time, *we fear*, before mothers know beforehand which of their sons will be surgeons and M.D.'s, and that, by strong imagination, these sons will in consequence be born with eyes in their fingers. . . . After all, (he avers,) the two stories have a good moral, even if not true; *but it is certainly fully within the bounds of rational probability*, that both are true." The force of faith could certainly no further go!

